# Efficacy of high-dose tigecycline-containing regimens for the combination treatment of carbapenem-resistant Enterobacterales bacteraemia

**DOI:** 10.1093/jacamr/dlaf162

**Published:** 2025-09-16

**Authors:** Worapong Nasomsong, Pattanapon Wanloptaree, Vasin Vasikasin

**Affiliations:** Division of Infectious Disease, Department of Internal Medicine, Phramongkutklao Hospital and Phramongkutklao College of Medicine, Bangkok, Thailand; Division of Infectious Disease, Department of Internal Medicine, Phramongkutklao Hospital and Phramongkutklao College of Medicine, Bangkok, Thailand; Division of Infectious Disease, Department of Internal Medicine, Phramongkutklao Hospital and Phramongkutklao College of Medicine, Bangkok, Thailand; Section of Adult Infectious Disease, Department of Infectious Disease, Faculty of Medicine, Imperial College London, London, UK

## Abstract

**Background:**

Carbapenem-resistant Enterobacterales (CRE) are critical priority pathogens due to limited therapeutic options. Standard-dose tigecycline (100 mg/day) may not achieve adequate plasma levels for treating CRE bacteraemia. High-dose tigecycline (HD-TGC; 200 mg/day) may improve drug exposure and clinical outcomes, though supporting evidence remains limited. This study aimed to evaluate the efficacy of an HD-TGC-containing regimen compared with other treatments for CRE bacteraemia.

**Methods:**

We conducted a retrospective propensity-score matched study at Phramongkutklao Hospital in Bangkok, Thailand, including adults with monomicrobial CRE bacteraemia (2017–2021). The primary outcome was 30-day all-cause mortality. Secondary outcomes were clinical and microbiological responses on Day 7.

**Results:**

Of 161 eligible patients, 37 (23.0%) received HD-TGC and 124 received other regimens. *Klebsiella pneumoniae* accounted for 90.6% of cases. Genotypic resistance testing in 28 isolates revealed *bla*_OXA-48_ (53.6%), *bla*_NDM_ (21.4%), and *bla*_NDM_ _+_ _OXA-48_ (21.4%) genes. The median age was 71 years (IQR 49–81). Patients in the HD-TGC group had more severe illness, including higher Pitt bacteraemia scores and vasopressor use. After 1:1 matching, 37 pairs were analysed. A non-significant trend towards lower 30-day mortality was observed in the HD-TGC group (HR 0.59; 95% CI: 0.30–1.14; *P* = 0.118). Microbiological response on Day 7 was significantly higher in the HD-TGC group (75.9% versus 46.2%; *P* = 0.027), while clinical response rates were not significantly different (48.6% versus 37.8%; *P* = 0.479).

**Conclusions:**

HD-TGC was associated with improved microbiological clearance and showed a trend towards reduced mortality in patients with CRE bacteraemia.

## Introduction

Carbapenem-resistant Enterobacterales (CRE) are clinically significant pathogens of global concern, contributing to increased morbidity, mortality, prolonged hospital stays and healthcare resource utilization.^1,2^ The therapeutic challenge of CRE stems from their resistance to multiple antibiotic classes, particularly carbapenems, which are often last-resort agents for Gram-negative infections.

Current treatment guidelines for CRE infections recommend individualized regimens based on the site of infection, antimicrobial susceptibility profiles, availability of first-line agents and the presence of specific carbapenemase genes.^3^ In particular, the therapeutic options for metallo-β-lactamase (MBL)-producing strains (e.g. *bla*_NDM_) are limited due to intrinsic resistance to novel β-lactam/β-lactamase inhibitor (BL–BI) combinations such as ceftazidime–avibactam, which are more effective against KPC and OXA-48-like producers.

Molecular surveillance in the Asia-Pacific region has demonstrated that *Klebsiella pneumoniae* harbouring *bla*_NDM_ predominates among CRE isolates.^4,5^ In Thailand, ceftazidime–avibactam is active against only approximately half of CRE isolates, limiting its utility.^6^ With the unavailability of aztreonam and limited efficacy of first-line agents, alternative or combination regimens—such as colistin, aminoglycosides, fosfomycin and tigecycline—are commonly employed. Monotherapy with these agents, however, has been associated with inferior outcomes compared with combination therapy involving at least one active agent.^7^

Tigecycline, which retains *in vitro* activity against CRE and has a favourable toxicity profile compared with polymyxins or aminoglycosides,^8,9^ is limited by its high volume of distribution and low plasma concentrations following standard dosing.^10^ In contrast, high-dose tigecycline (HD-TGC; 200 mg loading dose followed by 100 mg every 12 h) has been shown to achieve pharmacokinetic/pharmacodynamic (PK/PD) targets adequate for treating bloodstream infections and ventilator-associated pneumonia.^10,11^

Studies in extensively drug-resistant *Acinetobacter baumannii* infections suggest that HD-TGC improves clinical outcomes and minimizes toxicity.^12^ Meta-analyses further support the use of standard-dose tigecycline- or polymyxin-based combination regimens, which are associated with lower 30-day mortality compared with monotherapy, particularly in critically ill patients and those with CRE bacteraemia.^13–15^ A study found that HD-TGC was associated with significantly longer survival time in CRE bacteraemia as compared with standard dose.^16^ However, data specifically evaluating HD-TGC in CRE bacteraemia as compared with other best available regimens remain limited.

To address these gaps, this study aimed to evaluate the efficacy of HD-TGC in combination therapy compared with other best available regimens for the treatment of CRE bacteraemia, with the goal of informing more effective clinical management strategies.

## Material and method

### Study setting and population

This retrospective propensity score-matched study was conducted at Phramongkutklao Hospital, a 1200-bed tertiary university hospital in Bangkok, Thailand, from January 2017 to December 2021. Adult patients (≥20 years) with monomicrobial CRE bacteraemia were eligible for inclusion. Exclusion criteria included (i) transfer to another facility within 7 days of diagnosis, (ii) death within 48 h of initiating definitive antimicrobial therapy and (iii) allergy or intolerance [Common Terminology Criteria for Adverse Events (CTCAE) Grade ≥ 3] to study antibiotics. Demographic characteristics, clinical data, treatment regimens and outcomes were obtained from electronic medical records.

Patients in the HD-TGC group received definitive therapy comprising a 200 mg loading dose followed by 100 mg every 12 h, in combination with at least one agent demonstrating *in vitro* susceptibility. The comparator group received a combination of at least two active agents, excluding HD-TGC.

### Outcomes measurement

The primary outcome was 30-day all-cause mortality. Secondary outcomes included clinical and microbiological responses at Day 7. Clinical response was defined as improvement or resolution of infection-related signs and symptoms without new site involvement or the need for additional antimicrobial therapy. Microbiological response was defined as clearance of bacteraemia within 7 days of onset.

### Bacterial isolates and susceptibility testing

All CRE isolates were identified using matrix-assisted laser desorption ionization time-of-flight mass spectrometry (MALDI-TOF MS; MALDI Biotyper^®^, MA, USA). Antimicrobial susceptibility testing was performed via the broth microdilution method (Sensititre^™^, Thermo Fisher Scientific, MA, USA) and interpreted using European Committee on Antimicrobial Susceptibility Testing (EUCAST) criteria for tigecycline^17^ and Clinical and Laboratory Standards Institute (CLSI) guidelines.^18^ CRE was defined as Enterobacterales resistant to at least one carbapenem (imipenem, meropenem, doripenem, or ertapenem).^19^

### Statistical analysis

For categorical variables, chi-square or Fisher’s exact test was used. Student’s *t*-test or the Mann–Whitney *U* was used to compare continuous variables. To adjust for treatment selection bias, 1:1 propensity score matching was performed. Propensity scores were estimated using multivariable logistic regression including the following covariates: age, sex, INCREMENT-CPE score, Pitt bacteraemia score, presence of disseminated intravascular coagulation (DIC) and vasopressor use. Baseline characteristics were considered well balanced if standardized mean differences (SMDs) were <0.20. The Kaplan–Meier estimator was used to compute survival patterns, and the log rank test was used to compare survival between both groups. Microbiological response was evaluated only in patients who had follow-up blood cultures within the first 7 days. Patients who died during this period were classified as having no microbiological response. For all analyses, a two-sided *P* value of <0.05 was considered significant. Data were analysed using R software.

### Ethics considerations

This study was approved by the Institutional Review Board of the Royal Thai Army Medical Department (approval number: R081h/64_Exp), in accordance with international ethical guidelines including the Declaration of Helsinki, the Belmont Report, CIOMS Guidelines, and ICH-GCP. Given the retrospective design, a waiver of informed consent was granted.

## Results

### Baseline characteristic

**Figure 1.**
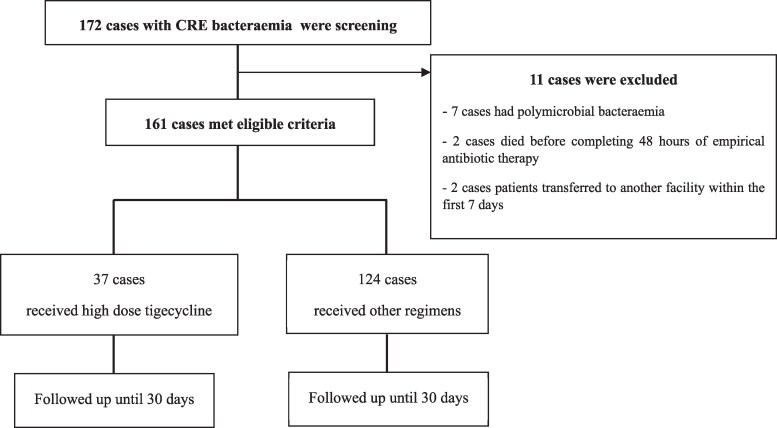
Enrolment diagram.

Between January 2017 and December 2021, a total of 172 patients were identified with CRE bacteraemia, of whom 161 met the inclusion criteria. Thirty-seven patients (23.0%) received HD-TGC, while 124 (77.0%) received alternative treatment regimens (Figure 1). *K. pneumoniae* was the predominant pathogen, accounting for 90.6% of isolates. Among isolates tested for tigecycline susceptibility, 25 of 26 (96.2%) in the HD-TGC group and 98 of 112 (87.5%) in the comparator group were found to be susceptible.

The three most common sources of bacteraemia in the HD-TGC group were intra-abdominal infections, primary bacteraemia and pneumonia. In contrast, primary bacteraemia, urinary tract infections and catheter-related bloodstream infections were most common in the comparator group. Genotypic resistance testing, performed on 28 available isolates (12 from the HD-TGC group and 16 from the comparator group), identified *bla_OXA-48_* (53.6%), *bla_NDM_* (21.4%) and co-harbouring *bla_NDM+OXA-48_* (21.4%).

The median age was 71 years (IQR: 49–81). Common comorbidities included hypertension, diabetes mellitus and chronic kidney disease. On the day of blood culture acquisition, more than half of the patients in both groups required intensive care unit admission, mechanical ventilation and vasopressor support. The prevalence of hyperlactatemia, respiratory failure, acute kidney injury and septic shock was similar between groups. However, the HD-TGC group had significantly higher rates of DIC, elevated Pitt bacteraemia scores and vasopressor use (Table 1).

**Table 1.** Baseline characteristics on the day of culture acquisition

Characteristic	Overall cohort			Propensity score-matched cohort		
	High-dose tigecycline (*n* = 37)	Others (*n* = 124)	*P* value	High-dose tigecycline (*n* = 37)	Others (*n* = 37)	*P* value
Male sex	25 (67.6)	79 (63.7)	0.667	25 (67.6)	27 (73.0)	0.813
Age (years)	71 (49–81)	66 (55–81)	0.641	71 (49–81)	76 (63–83)	0.174
*Klebsiella pneumoniae* infections	36 (97.3)	109 (87.9)	0.122	36 (97.3)	36 (97.3)	1.000
Intensive care unit admission	24 (64.9)	63 (50.8)	0.188	24 (64.9)	22 (59.5)	0.811
Underlying diseases						
Diabetes	13 (35.1)	41 (33.1)	0.815	13 (35.1)	17 (45.9)	0.476
Hypertension	21 (56.8)	70 (56.5)	0.974	21 (56.8)	24 (64.9)	0.645
Cirrhosis	3 (8.1)	12 (9.7)	1.000	3 (8.1)	4 (10.8)	1.000
Chronic kidney diseases	12 (32.4)	27 (21.8)	0.184	12 (32.4)	7 (18.9)	0.295
Congestive heart failure	3 (8.1)	14 (11.3)	0.764	3 (8.1)	4 (10.8)	1.000
Chronic lung diseases	2 (5.4)	6 (4.8)	1.000	2 (5.4)	2 (5.4)	1.000
Biliary stone	1 (2.7)	10 (8.1)	0.459	1 (2.7)	2 (5.4)	1.000
Autoimmune diseases	3 (8.1)	7 (5.6)	0.698	3 (8.1)	4 (10.8)	1.000
Cerebrovascular disease	7 (18.9)	20 (16.1)	0.690	7 (18.9)	10 (27.0)	0.591
Dementia	3 (8.1)	7 (5.6)	0.698	3 (8.1)	4 (10.8)	1.000
Solid malignancy	7 (18.9)	25 (20.2)	0.868	7 (18.9)	5 (13.5)	0.748
Haematologic malignancy	8 (21.6)	23 (18.5)	0.677	8 (21.6)	9 (24.3)	1.000
HIV	1 (2.7)	3 (2.4)	1.000	1 (2.7)	1 (2.7)	1.000
Transplant	2 (5.4)	3 (2.4)	0.324	2 (5.4)	0 (0.0)	0.488
Immunosuppressive	14 (37.8)	35 (28.2)	0.265	14 (37.8)	11 (29.7)	0.644
Pressure sore	5 (13.5)	11 (8.9)	0.407	5 (13.5)	3 (8.1)	0.709
Smoking	1 (2.7)	7 (5.6)	0.683	1 (2.7)	4 (10.8)	0.362
Alcohol	1 (2.7)	7 (5.6)	0.683	1 (2.7)	4 (10.8)	0.375
Clinical parameters						
Lactate (>2 mmol/L)	25/36 (69.4)	71/124 (57.3)	0.189	25/36 (69.4)	27/37 (73.0)	0.801
Respiratory failure	27 (73.0)	80 (64.5)	0.339	27 (73.0)	27 (73.0)	1.000
Acute kidney injury	31 (83.8)	90 (72.6)	0.166	31 (83.8)	31 (83.8)	1.000
DIC	26 (70.3)	58 (46.8)	0.012	26 (70.3)	26 (70.3)	1.000
Pitt Bacteraemia score ≥ 6	22 (59.5)	43 (34.7)	0.007	22 (59.5)	19 (51.4)	0.638
Central venous catheter	19 (51.4)	62 (50.0)	0.885	19 (51.4)	23 (62.2)	0.453
Arterial catheter	17 (45.9)	43 (34.7)	0.213	17 (45.9)	18 (48.6)	1.000
Foley catheter	24 (64.9)	83 (66.9)	0.815	24 (64.9)	28 (75.7)	0.446
Cardiac arrest	2 (5.4)	3 (2.4)	0.324	2 (5.4)	1 (2.7)	1.000
Ventilator	27 (73.0)	79 (63.7)	0.297	27 (73.0)	29 (78.4)	0.796
Vasopressor	22 (59.5)	50 (40.3)	0.040	22 (59.5)	21 (56.8)	1.000
Endotracheal intubation	26 (70.3)	81 (65.3)	0.693	26 (70.3)	30 (81.1)	0.409
Charlson Comorbidity Index	2 (2–4)	2 (2–4)	0.992	2 (2–4)	3 (2–4)	0.655
INCREMENT-CPE score	14 (8–15)	10 (6–14)	0.061	14 (8–15)	14 (8–17)	0.586
Source of bacteraemia			0.273			0.566
Intra-abdominal infection	10 (27.0)	18 (14.5)		10 (27.0)	5 (13.5)	
CRBSI	5 (13.5)	22 (17.7)		5 (13.5)	6 (16.2)	
Febrile neutropenia	3 (8.1)	7 (5.6)		3 (8.1)	2 (5.4)	
Pneumonia	7 (18.9)	19 (15.3)		7 (18.9)	7 (18.9)	
SSTI	3 (8.1)	4 (3.2)		3 (8.1)	2 (5.4)	
UTI	2 (5.4)	24 (19.4)		2 (5.4)	6 (16.2)	
Primary bacteraemia	7 (18.9)	30 (24.2)		7 (18.9)	9 (24.3)	

CRBSI, catheter-related bloodstream infections; CPE, carbapenemase-producing Enterobacterales; DIC, disseminated intravascular coagulation; SSTI, skin and soft tissue infections; UTI, urinary tract infection.

In the HD-TGC group, aminoglycosides (67.6%) and colistin (40.5%) were the most frequently used combination agents. In the comparator group, commonly used agents included colistin (54.0%), fosfomycin sodium (42.8%), high-dose carbapenems given as extended infusion (37.8%) and aminoglycosides (24.2%) (Table 2).

**Table 2.** Antibiotic profiles

Antibiotics	Overall cohort		Propensity score-matched cohort	
	High-dose tigecycline (*n* = 37)	Others (*n* = 124)	High-dose tigecycline (*n* = 37)	Others (*n* = 37)
Time to definitive treatment (days)	3 (2–4)	3 (2–4)	3 (2–4)	2 (2–3.5)
Tigecycline (high dose)	37 (100)	0 (0)	37 (100)	0 (0)
Tigecycline (standard dose)	0 (0)	7 (5.7)	0 (0)	3 (8.1)
Colistin	15 (40.5)	67 (54)	15 (40.5)	21 (56.8)
Aminoglycoside	25 (67.6)	70 (56.5)	25 (67.6)	22 (59.5)
Fosfomycin sodium	0 (0)	53 (42.8)	0 (0)	18 (48.6)
High dose imipenem	0 (0)	19 (15.3)	0 (0)	3 (8.1)
High dose meropenem	0 (0)	25 (20.2)	0 (0)	10 (27.0)
Others	3 (8.1)	7 (5.6)	3 (8.1)	0 (0)

### Primary and secondary outcomes

The 30-day all-cause mortality was 40.5% in the HD-TGC group and 35.5% in the comparator group (*P* = 0.775). Clinical response rates at Day 7 were 48.6% and 56.5% in the HD-TGC and comparator groups, respectively (*P* = 0.403), and at Day 14 were 37.8% and 52.4%, respectively (*P* = 0.119). Follow-up blood cultures within the first 7 days were performed in 28 patients (75.7%) in the HD-TGC group and 78 patients (62.9%) in the comparator group. Microbiological response at Day 7 was 75.9% in the HD-TGC group versus 65.9% in the comparator group (*P* = 0.379).

**Figure 2.**
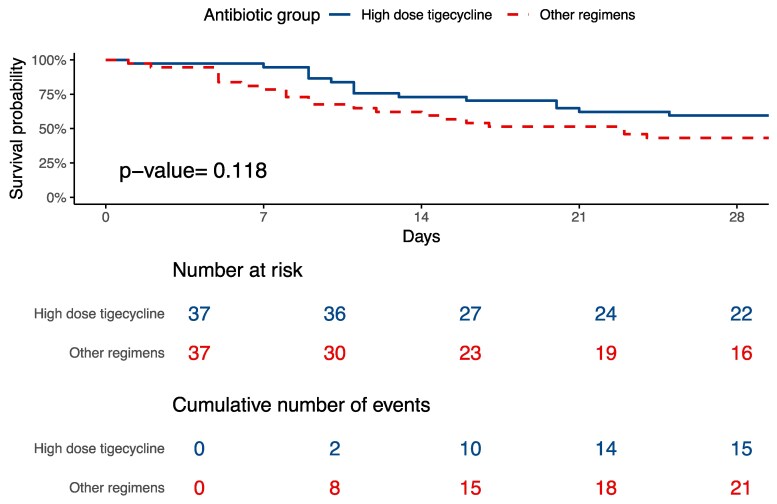
The Kaplan–Meier survival analysis of 30-day all-cause mortality between HD-TGC and others group among the propensity score-matched cohort.

Propensity score matching yielded 37 pairs of patients. Among isolates tested for tigecycline susceptibility, 25 of 26 (96.2%) in the HD-TGC group and 30 of 33 (90.9%) in the matched comparator group were susceptible. Following matching, a trend towards lower 30-day mortality was observed in the HD-TGC group compared with the comparator group [40.5% versus 71.4%, hazard ratio (HR) 0.59; 95% CI: 0.30–1.14; *P* = 0.118]. Clinical response on Day 7 was 48.6% in the HD-TGC group versus 37.8% in the comparator group (*P* = 0.474). Follow-up blood cultures within 7 days were obtained in 28 patients (75.7%) and 22 patients (59.5%), respectively. Microbiological response on Day 7 was significantly higher in the HD-TGC group (75.9%) compared with the comparator group (46.2%; *P* = 0.027) (Table 3). Kaplan–Meier survival analysis comparing 30-day mortality between groups is shown in Figure 2.

**Table 3.** Outcomes

Characteristic	Overall cohort			Propensity score-matched cohort		
	High-dose tigecycline (*n* = 37)	Others (*n* = 124)	*P* value	High-dose tigecycline (*n* = 37)	Others (*n* = 37)	*P* value
Outcomes						
Clinical response at Day 7	18 (48.6)	70 (56.5)	0.403	18 (48.6)	14 (37.8)	0.479
Clinical response at Day 14	14 (37.8)	65 (52.4)	0.119	14 (37.8)	13 (35.1)	1.000
Microbiological response at Day 7	22/29 (75.9)	56/85 (65.9)	0.379	22/29 (75.9)	12/26 (46.2)	0.027
30-Day mortality	15 (40.5)	44 (35.5)	0.775	15 (40.5)	21 (71.4)	0.118

## Discussion

In this propensity score-matched retrospective cohort study, HD-TGC in combination with aminoglycosides or colistin for the treatment of CRE bacteraemia was associated with a trend towards reduced 30-day all-cause mortality compared with other combination regimens, although the difference did not reach statistical significance. The 30-day mortality rate in the HD-TGC group was 40.5%, consistent with previous studies reporting mortality rates ranging from 27.3% to 52.2% in patients treated with tigecycline-based regimens.^14,20–23^ In contrast, the comparator group exhibited a notably higher mortality rate (71.4%), which may reflect the higher baseline severity of illness in this cohort.

Despite the observed benefit of HD-TGC-based therapy, the 40.5% mortality rate remains unacceptably high, highlighting the overall poor prognosis of patients with CRE bacteraemia. Several factors may contribute to this persistently high mortality. First, patients with CRE infections often present with significant comorbidities, immunosuppression or critical illness, all of which are independently associated with worse outcomes as shown in the baseline characteristics of this study. Second, delays in initiating effective antimicrobial therapy due to diagnostic challenges or limited susceptibility data may compromise clinical response. Third, access to newer and more potent agents—such as ceftazidime–avibactam or meropenem–vaborbactam—may be restricted in some settings due to availability, cost or genotypic susceptibility. These findings emphasize the need for novel agents and improved strategies for antimicrobial optimization in managing CRE bacteraemia.

Factors known to contribute to poor outcomes in CRE bacteraemia—including the presence of septic shock and acute respiratory failure at onset—were common in both groups and are consistent with prior findings.^20,21,24^ Notably, rates of DIC, elevated Pitt bacteraemia scores and vasopressor use were significantly higher in the HD-TGC group, reflecting the greater severity of illness in this cohort. These factors were used for propensity score matching, resulting in a less biased comparison.

The clinical response rates on Days 7 and 14 in the HD-TGC group were 48.6% and 37.8%, respectively, which are comparable to those reported in previous studies.^14^ Microbiological response at Day 7 was significantly higher in the HD-TGC group (75.9%) compared with the comparator group (46.2%), aligning with prior reports indicating microbiological eradication rates of approximately 51.8% with tigecycline-based therapy.^14^ The comparatively lower response rates observed in the comparator group may reflect the reduced efficacy of alternative regimens in this patient population.

Several factors may explain the modest efficacy of the comparator regimens. First, patients in this cohort had high baseline disease severity, as evidenced by elevated INCREMENT-CPE scores.^25^ Second, all treatment regimens employed were considered alternative therapies per international guidelines, due to the limited availability of newer agents and the predominance of *bla*_NDM_-producing isolates in the region.^3,26^ Among the comparator group, high-dose carbapenems, used in 37.8% of patients, have previously demonstrated modest clinical efficacy (29%–69%) in CRE infections.^27^ Similarly, while fosfomycin sodium has shown potential activity *in vitro* and in limited clinical series, its efficacy in CRE bacteraemia remains uncertain.^28,29^ One study suggested a non-significant survival benefit when fosfomycin was used in combination therapy for CRE bacteraemia, following adjustment for confounders.^30^ Finally, the PK and PD of critically ill patients may be altered. In some studies, *C*_max_ and AUC were found to be relatively low in critically ill patients, even when HD-TGC were administered.^21^

To our knowledge, this study is among the few evaluating HD-TGC in a setting with high prevalence of *bla*_NDM_ and *bla*_OXA-48_-like carbapenemase genes, where therapeutic options remain particularly constrained. However, this study has several limitations. First, the sample size was limited, potentially affecting statistical power. Second, despite the use of propensity score matching, residual confounding cannot be excluded due to the retrospective observational design. Third, molecular characterization of resistance mechanisms and virulence factors was not performed in all isolates, which may influence treatment outcomes. Fourth, we did not systematically assess the dosing of agents other than tigecycline. Nevertheless, the presence of an active infectious disease pharmacy team likely ensured appropriate dose adjustments based on renal function and haemodynamic status. Finally, follow-up blood cultures were not uniformly collected—8 of 37 patients (21.6%) in the HD-TGC group and 11 of 37 (29.7%) in the comparator group lacked follow-up cultures—which could introduce bias in the microbiological response comparison.

Future research should involve prospective, multicentre studies with larger sample sizes and PK/PD monitoring to validate these findings and explore optimal dosing strategies. Additionally, further studies should focus on the efficacy of HD-TGC in combination with newer agents or under combination regimens guided by molecular resistance profiles.

In conclusion, HD-TGC was associated with improved microbiological responses and a non-significant trend towards lower 30-day mortality in patients with CRE bacteraemia. In regions where access to newer antimicrobial agents is limited, HD-TGC in combination with other active agents may represent a viable alternative for the treatment of CRE bacteraemia.
